# Immunoengineering strategies using nanoparticles for obesity treatment

**DOI:** 10.26599/NR.2025.94907707

**Published:** 2025-12

**Authors:** Neda Mohaghegh, Narges Zargar Balajam, Bahareh Hosseinpour, Claire Buttles, Qiang Huang, Yixuan Huang, Amir Ahari, Neda Farhadi, Saya Davani, Safoora Khosravi, Bahareh Mirmashhouri, Negar Hosseinzadeh Kouchehbaghi, Rohan Sampath, Mohsen Akbari, Vadim Jucaud, Heemin Kang, Ali Khademhosseini, Ryan M. Pearson, Alireza Hassani Najafabadi

**Affiliations:** 1 Terasaki Institute for Biomedical Innovation, Los Angeles, CA 91367, USA; 2 Department of Chemistry, Carleton University, Ottawa, ON K1S 5B6, Canada; 3 Indiana University Bloomington, Department of Biology, Bloomington, IN 47405, USA; 4 Department of Neurobiology, Physiology, and Behavior, University of California Davis, Briggs Hall, Davis, CA 95616, USA; 5 Electrical and Computer Engineering Department, University of British Columbia, Vancouver, BC V6T 1Z4, Canada; 6 Laboratory for Innovations in MicroEngineering (LiME), Department of Mechanical Engineering, University of Victoria, Victoria, BC V8P 5C2, Canada; 7 Materials Science and Engineering, Korea University, Seoul 02841, Republic of Korea; 8 Department of Pharmaceutical Sciences, University of Maryland School of Pharmacy, Baltimore, MD 21201, USA

**Keywords:** obesity, nanoparticles, inflammation, macrophage, immunomodulation

## Abstract

Obesity has emerged as a global epidemic, posing severe challenges to public health and contributing to various complications, including metabolic disorders, cardiovascular disease, and type 2 diabetes. This review provides a comprehensive overview of obesity, its associated comorbidities, and the limitations of conventional treatments. We explore the complex relationship between obesity-induced inflammation, immune dysregulation, and the pivotal role of adipose tissue macrophages (ATMs). Chronic low-grade inflammation in adipose tissues (AT) is a key driver of insulin resistance and metabolic dysfunction. As ATs expand, they undergo significant changes, including increased immune cell infiltration, particularly macrophages (MΦs), which shift from an anti-inflammatory towards a pro-inflammatory phenotype. This review aims to advance the understanding of immunomodulatory strategies by examining MΦ polarization and AT browning as promising therapeutic approaches. We focus on nanoparticles (NPs)-based strategies for immunomodulation, highlighting innovative engineering approaches designed to target the inflammatory pathways underlying obesity. By addressing these mechanisms, this review provides valuable insights into mitigating obesity-associated inflammation and related metabolic disorders, paving the way for novel therapeutic strategies in the fight against the global obesity epidemic.

## Graphical Abstract

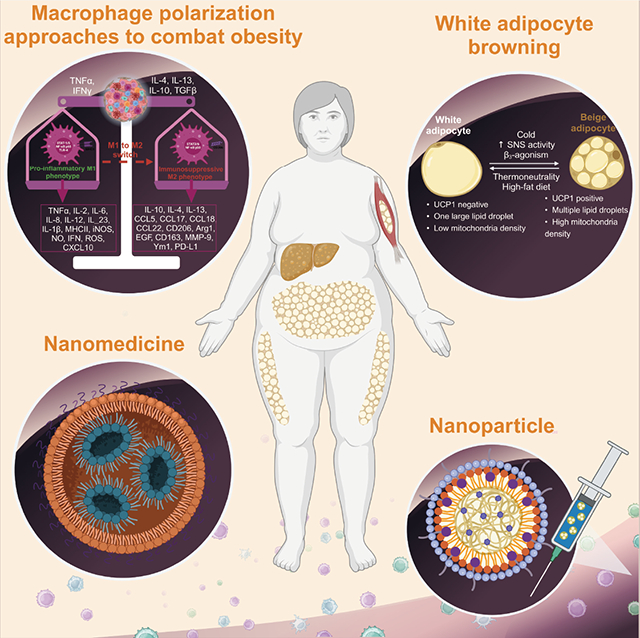

## 1 Introduction

Obesity has become a multifaceted epidemic affecting individuals of all ages, genders, and socioeconomic backgrounds. According to the World Health Organization (WHO), obesity is an abnormal or excessive accumulation of fat that poses a health risk [1]. With nearly 64% of the US population anticipated to be overweight or obese by 2025, the concern for related health risks has risen exponentially [2]. It stands as a significant risk factor for a range of metabolic diseases, including cardiovascular, type 2 diabetes (T2D), metabolic dysfunction-associated steatohepatitis (MASH), atherosclerosis, and cancer [3, 4]. Obesity is a complex disease that necessitates a multifaceted approach for successful clinical management. Treatment strategies can be broadly categorized into lifestyle changes and medical interventions [5–7]. Lifestyle changes encompass nutrition therapy, physical activity, and behavioral modifications. Medical interventions can include surgery or pharmacological management [8]. Despite the treatment options available, achieving and sustaining long-term weight reduction remains a critical challenge that will require innovative solutions to enhance patient outcomes [9].

The multifaceted nature of obesity underscores the importance of employing a comprehensive approach involving lifestyle changes coupled with medical interventions when necessary to yield sustainable improvements in overall health. Nutritional interventions have shown some benefits, but they are often insufficient alone unless paired with exercise regimens [10, 11]. Behavioral modification techniques can help individuals develop sustainable habits and coping mechanisms to overcome psychological barriers. Cognitive behavioral therapy (CBT) has emerged as a promising approach targeting behavioral and psychological factors influencing eating habits to promote long-term behavior change. Large-scale CBT implementation has proven difficult, and its long-term success has been unsatisfactory [12, 13]. Considering the significant challenges that patients must overcome to develop new habits, psychological factors, social and environmental influences, and other barriers, medical interventions may become necessary to enable obese patients to achieve and sustain meaningful weight loss and improve overall health outcomes [12, 14, 15].

Bariatric surgery is a procedure that has shown benefits in treating obese patients by reducing comorbidities and promoting weight loss [12, 16, 17]. Procedures like Roux-en-Y gastric bypass (RYGB) and gastric banding have also shown improved outcomes compared to other non-surgical interventions. RYGB is one of the most frequently performed bariatric surgeries worldwide and is considered the gold standard in surgical weight-loss interventions [18]. Despite the numerous advantages attributed to bariatric surgery, follow-up is necessary for the rest of a patient’s life to avoid any complications arising from the surgery, and adherence to recommended lifestyle changes is crucial for maintaining the benefits of bariatric surgery and preventing weight regain or other adverse effects [19].

The pharmacological management of obesity has witnessed significant advancements in recent years, offering promising alternatives beyond surgical approaches [20]. The Food and Drug Administration (FDA) has approved seven medications for the management of obesity: orlistat (Xenical, Alli), phentermine-topiramate (Qsymia), naltrexone-bupropion (Contrave), liraglutide (Saxenda), semaglutide (Wegovy), setmelanotide (Imcivree), and tirzepatide (Zepbound), which are summarized in Table 1 [21]. However, despite this progress, the field has also faced numerous setbacks, with several drugs being withdrawn from the market due to their adverse effects, such as risks to cardiovascular health, increased suicidal tendencies, drug dependence, and potential for abuse, among several others [22–24].

While obesity is commonly attributed to excessive caloric intake and a sedentary lifestyle, it is increasingly recognized as a chronic low-grade inflammatory condition driven by metabolic disturbances—a phenomenon known as metaflammation [25–27]. The recognition of metaflammation as a key factor in obesity challenges the traditional view and highlights the complex interplay between inflammation, immune dysregulation, and metabolic disturbances [28, 29]. Furthermore, understanding the mechanisms driving inflammation in obesity is crucial for developing effective therapeutic strategies to manage weight gain and improve metabolic health. This includes exploring innovative approaches that integrate immunomodulatory therapies tailored to individual genetic and metabolic profiles, leveraging emerging technologies and personalized medicine [30–32]. By leveraging these cutting-edge approaches, there is potential to develop innovative strategies to address the multifaceted nature of the condition, offering personalized solutions that account for the underlying physiological, genetic, and metabolic factors contributing to obesity [33].

This review will explore the understanding of obesity as an inflammatory condition, highlighting ATMs as key immune cells that contribute to metabolic dysfunction in obesity. MΦ polarization state, cytokines, and inflammatory signaling pathways in ATs as potential targets will also be discussed. Lastly, we will describe emerging nanoparticle (NP)-based approaches to achieve local and/or systemic immunomodulation in obesity, with a focus on lesser-studied MΦ-targeted therapies. By targeting the underlying inflammatory processes, these innovative approaches hold promises to effectively manage obesity and tackle its associated metabolic complications.

## 2 Fundamental role of adipocytes and macrophages (MΦs) in obesity

**Figure 1:**
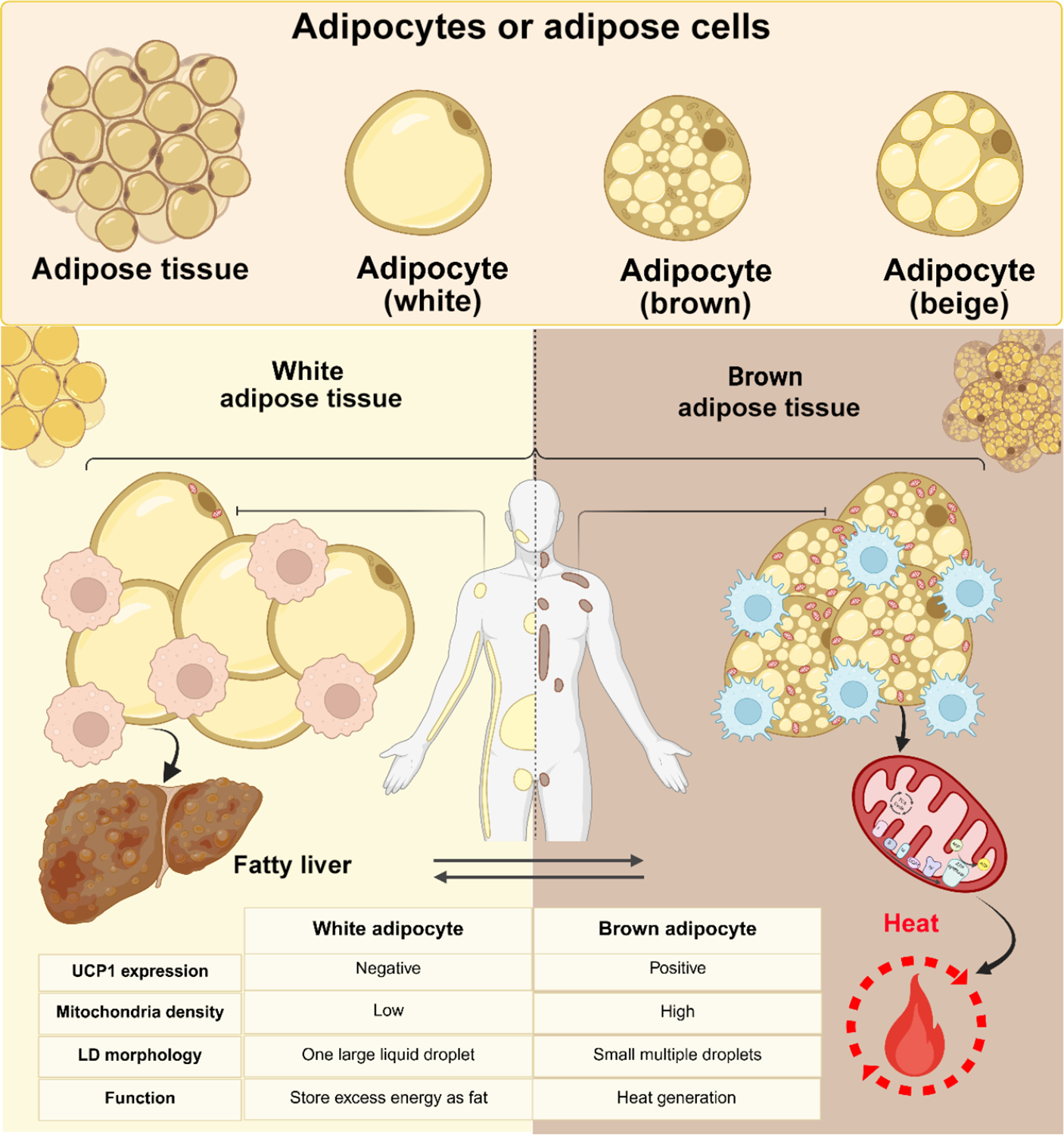
Exploring the dynamic roles and therapeutic potential of brown, beige, and white adipose tissue: BAT is primarily involved in thermogenesis, utilizing energy substrates to generate heat, a process facilitated by UCP1 in mitochondria. In contrast, WAT serves as the main site for energy storage. The browning process, which induces the formation of beige or brite adipocytes within WAT, enhances energy expenditure and holds promise as a therapeutic target for obesity. LD refers to lipid droplets integral to these tissues’ storage and metabolic functions (created by BioRender, adapted from “Adipose Tissue Depots”, by BioRender.com (2025). Retrieved from https://app.biorender.com/biorender-templates).

Obesity results from the excessive accumulation of ATs, specifically white adipose tissue (WAT), leading to a prolonged energy imbalance [34, 35]. Generally, adipocytes were classified into two main types based on their morphology, location, and function: white and brown adipocytes (Fig. 1) [36]. However, this classification has expanded to include a third category known as beige or brite (brown-in-white) adipocytes [37]. White adipocytes are typically large, round cells characterized by a prominent unilocular lipid droplet surrounded by a thin layer of cytoplasm that contains a few mitochondria. Their primary functions include lipolysis, energy storage in the form of fat, and adipokine secretion [38]. WATs serve as a mammal’s primary adipose organ, secreting various bioactive compounds that impact overall body function [39, 40].

Beige adipocytes are a distinct population of thermogenic fat cells that emerge within WAT in response to specific stimuli, such as chronic cold exposure, β3-adrenergic agonists, or certain pharmacological agents [41–43]. Morphologically and functionally, beige adipocytes resemble brown adipocytes, characterized by multilocular lipid droplets, high mitochondrial content, and robust expression of uncoupling protein 1 (UCP1), which enables them to dissipate energy as heat through non-shivering thermogenesis [41, 42].

In contrast, brown adipocytes within brown adipose tissues (BATs) are polygonal cells with multilocular lipid droplets and numerous mitochondria. Their main biological function is thermogenesis, although they also store energy as fat and secrete adipokines, albeit to a lesser extent than white adipocytes [37]. The mitochondria in brown adipocytes contain UCP1, which dissipates energy as heat by uncoupling respiration from adenosine triphosphate (ATP) synthesis [44].

The growth of ATs is a tightly regulated biological process influenced by hypertrophy, the increase in the size of existing adipocytes, and hyperplasia, the increase in cell number through the differentiation of preadipocytes into new adipocytes [45–47]. The number of adipocytes is largely established early in life, particularly after birth and adolescence, and remains relatively stable throughout adulthood. Adipocyte turnover is maintained by balancing adipogenesis, the formation of new adipocytes, with apoptosis, the programmed cell death of adipocytes [48]. White adipocytes are unique in their ability to grow significantly larger, primarily due to their role in storing excess fat as lipid droplets. This growth capacity is partly attributed to the inflammatory microenvironment within ATs, triggered by inflammatory signals that promote adipocyte hypertrophy, contributing to fat accumulation and WAT expansion [49–52]. This phenomenon underscores the complex interplay between immune cells as the primary sources of inflammation and AT in obesity and metabolic health [53, 54].

Various immune cells home to ATs and play pivotal roles in maintaining tissue homeostasis and regulating inflammatory processes, including MΦs, endothelial cells, and fibroblasts [55]. Among the immune cells in ATs, ATMs form the largest population, comprising 5%–10% of cells in lean ATs and increasing to 50% or more in cases of extreme obesity. This significant presence underscores their critical roles in obesity and metabolic inflammation [56, 57]. ATMs regulate physiological processes such as tissue remodeling, angiogenesis, and insulin sensitivity. MΦs are generally classified into two phenotypes: M1-like (M1) and M2-like (M2), characterized as pro-inflammatory (classically-activated) or anti-inflammatory (alternatively-activated), respectively. M1 MΦs secrete pro-inflammatory cytokines, such as tumor necrosis factor-alpha (TNF-α), interleukin-6 (IL-6), and interleukin-1 beta (IL-1β), which impair insulin signaling in adipocytes and contribute to insulin resistance. Conversely, M2 MΦs secrete anti-inflammatory cytokines, such as interleukin-10 (IL-10) and transforming growth factor beta (TGF-β), which maintain functional insulin signaling and promote tissue repair and remodeling [57–59].

The complex interaction between obesity, inflammation, and the immune system, particularly the role of MΦs in ATs, underscores the potential of immunomodulatory strategies as promising avenues for developing novel and effective treatments for this complex metabolic disorder [3, 60]. By modulating the inflammatory response within ATs and restoring the balance between pro- and anti-inflammatory immune cells, immunomodulatory therapies may offer a targeted approach to mitigating the harmful effects of chronic inflammation associated with obesity and its related comorbidities [61, 62]. Furthermore, immunomodulatory approaches can be combined with other obesity countermeasures, such as lifestyle interventions, pharmacotherapy, or bariatric surgery, to enhance their effectiveness and address the underlying inflammatory processes. Table 2 provides an overview of major inflammatory and metabolic markers that reflect the complex immune environment of adipose tissues. In the following section, we will discuss the mechanisms by which the immune system contributes to obesity and explore how immunotherapy treatment uses NPs to address this issue.

### 2.1 MΦ polarization, inflammation, and its effects in lean and obese ATs

Research indicates that obesity-induced changes in MΦ populations and phenotypes lead to chronic inflammation and insulin resistance. In obese individuals, ATs shift towards a pro-inflammatory state characterized by increased recruitment and accumulation of M1 MΦs [63, 64]. These MΦs secrete pro-inflammatory cytokines such as TNF-α, IL-6, and IL-1β, which inhibit insulin signaling in adipocytes and promote insulin resistance. Additionally, obesity causes adipocyte death and dysfunction, further fueling inflammation [65]. The clearance of dead adipocytes by MΦs may promote their pro-inflammatory activation, perpetuating chronic inflammation and metabolic dysregulation, crucial risk factors for insulin resistance and type 2 diabetes [66].

**Figure 2:**
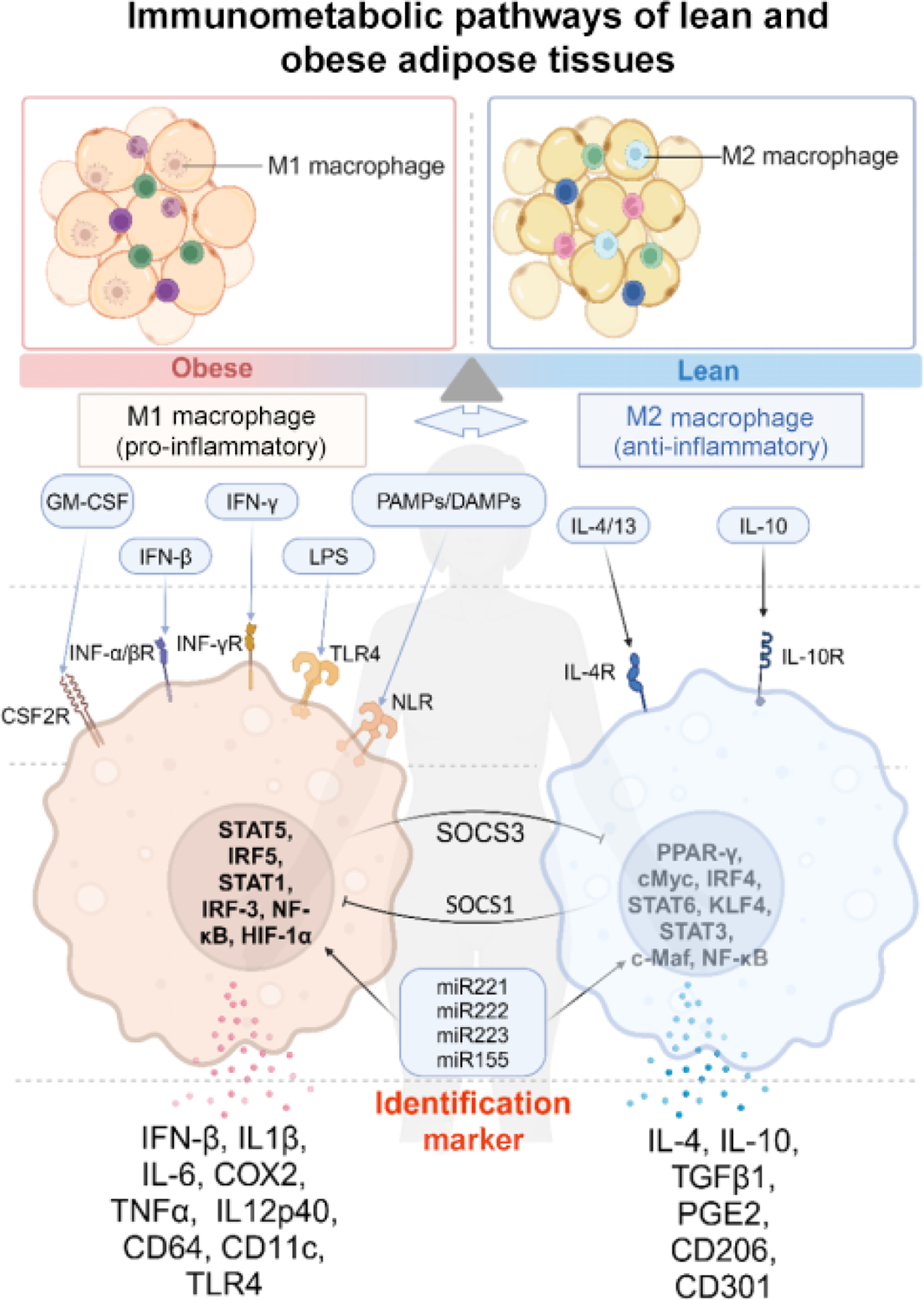
Mechanism of MΦ polarization. The immunometabolic state of ATs is distinctly different between lean and obese conditions. In lean AT, adipocytes secrete anti-inflammatory cytokines. Resident M2-like ATMs maintain an anti-inflammatory environment by producing factors like IL-10 and arginase. In obesity, adipocyte hypertrophy and hyperplasia increase pro-inflammatory cytokine secretion. This promotes recruitment and M1 polarization of pro-inflammatory ATMs. The balance between pro- and anti-inflammatory signals from adipocytes and ATMs in the AT microenvironment dictates the overall immunometabolic state and metabolic dysfunction associated with obesity (created by BioRender, adapted from “Macrophage Polarization: M1 and M2 Subtypes”, by BioRender.com (2025). Retrieved from https://app.biorender.com/biorender-templates).

MΦs, as mononuclear phagocytes, maintain tissue homeostasis by scavenging debris, pathogens, and necrotic or apoptotic cells. They adapt quickly to environmental stimuli through cell surface receptors like Toll-like receptors (TLRs), which trigger transcriptional programs when bound to damage- or pathogen-associated molecules. In the shift from lean to obese ATs, cytokine signaling and MΦ polarization and metabolism change. In lean ATs, MΦs account for about 10%–15% of cells, while in obese ATs, this increases to 40%–60% [63]. In lean tissue, MΦs perform efferocytosis, lipid buffering, and angiogenesis tasks. M2 MΦs interact with preadipocytes, releasing factors that enhance survival and angiogenesis [67–69]. Although MΦ phenotypes exist on a spectrum [70], they can generally be classified as pro-inflammatory, M1, or anti-inflammatory, M2 states [71–73], each with distinct roles in maintaining local microenvironments and inflammation levels (Fig. 2). In response to excessive AT expansion, the balance of MΦ phenotypes in ATs shifts from 4:1 (lean) to 1.2:1 (obese) M2-to-M1 ratio [71], while enhancing the trafficking of additional immune cells into fat tissue [74]. Taken together, the alteration of MΦ polarization contributes to the transformation of BATs into WATs, a hallmark of obesity [58, 75, 76].

Monocyte-derived MΦ infiltration into ATs during obesity has been observed in both mice and humans, originating from bone marrow due to increased blood monocyte diapedesis [63, 77]. Surgical weight loss reduces MΦ infiltration in obese patients. Obese mice on a high-fat diet develop M1 MΦs in ATs, increasing inflammatory gene expression and reducing IL-10, thus altering the M2-to-M1 ratio [64, 78–83]. Chemokines and their receptors, like MCP-1 and CCR2, play roles in attracting monocytes to AT in obesity. Elevated MCP-1 levels in obesity lead to increased MΦ infiltration and metabolic disruption. MΦ accumulation in AT correlates with body fat markers, and recent studies suggest that this may stabilize at higher adiposity levels or be affected by leptin deficiency, particularly in the subcutaneous depot [84–89].

One of the key pathways regulating MΦ polarization is the IFN regulatory factor/signal transducer and activator of the transcription (IRF/STAT) signaling pathway [90]. MΦs can be driven towards a potential M1 phenotype through TLR signaling, which is activated by lipopolysaccharides (LPS) and other microbial ligands [91]. This pathway is mediated by the MyD88 adaptor protein that activates a series of kinases, including TRAF6, IRAK4, and IKKβ, ultimately activating nuclear factor kappa B (NF-κB). As a central transcription factor, NF-κB promotes M1 polarization by inducing the expression of numerous inflammatory genes, such as *IL1β*, *IL-6*, *COX2*, *TNF-α*, and *IL12p40* [92]. Additionally, the resulting expression of IRF3 and IRF5 through the TRIF adaptor pathway is also widely reported to be related to M1 polarization and M1-associated gene induction [93]. Other mechanisms, such as hypoxia and adipocyte death, have been implicated in MΦ-driven inflammation in AT, particularly when a clear initiating trigger is lacking.

The IRF/STAT signaling pathway plays a significant role in modulating M1–M2 polarization in ATMs [92]. When exposed to various environmental stimuli, such as IFN, LPS, IL-4, and IL-10, MΦs activate different receptors, leading to STAT and IRF signaling cascades. The balance between STAT1 (pro-inflammatory) and STAT3/STAT6 (anti-inflammatory) determines whether MΦs adopt an M1 or an M2 phenotype. Additionally, M1 and M2 MΦ populations can inhibit each other by expressing SOCS1 and SOCS3, which suppress opposing STAT pathways [26]. MicroRNAs (miR221–223 and miR155) have been shown to regulate the signaling pathway through both direct and indirect mechanisms. TLR4, which serves as both a receptor for LPS and a downstream product of M1 activation, reinforces M1 polarization, while IL-4 and IL-10 promote M2 differentiation [26, 94].

**Figure 3:**
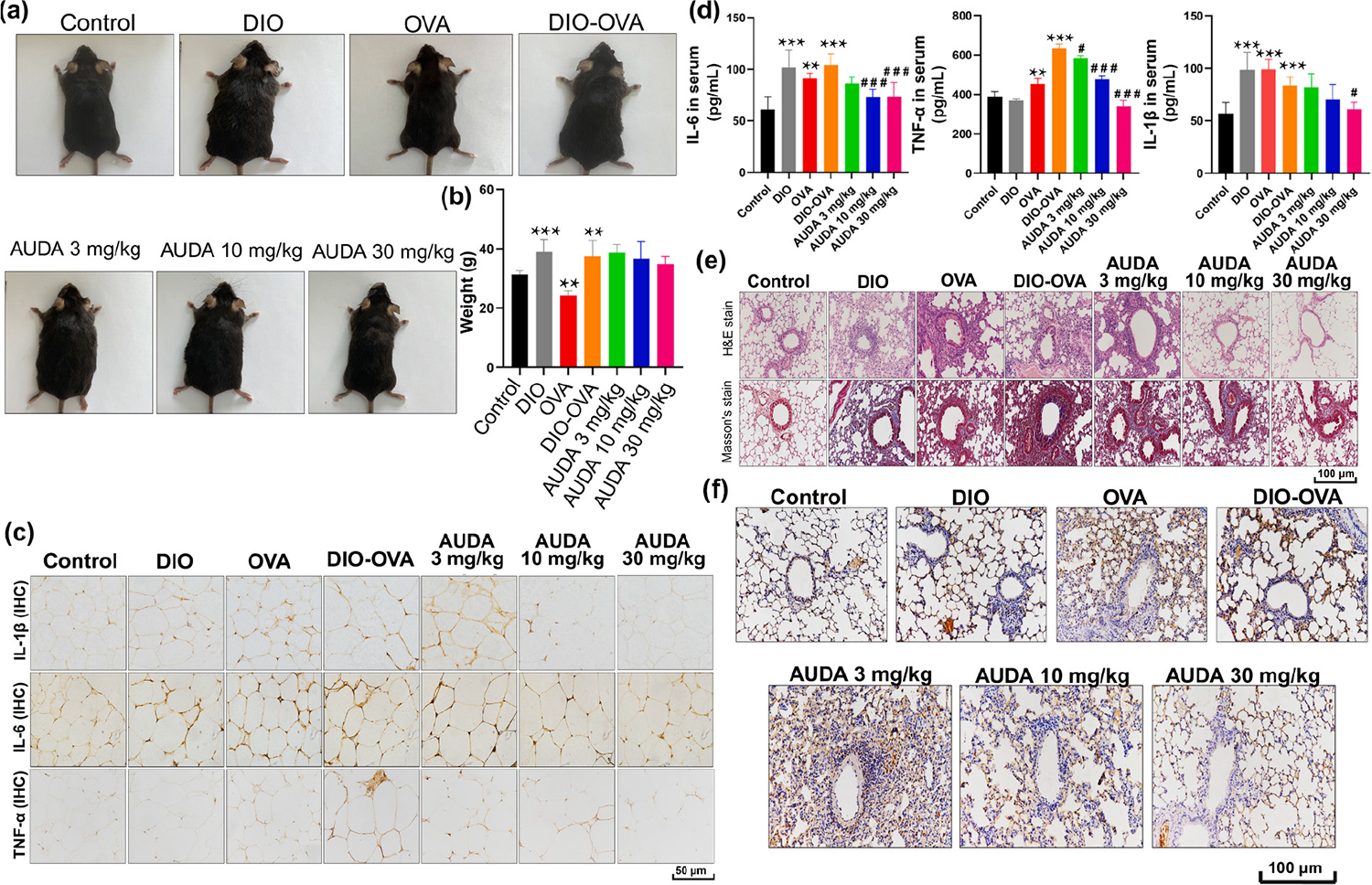
Modulation of ATM polarization by soluble epoxide hydrolase inhibition in obese asthma. (a) Illustrations and photographs comparing mice treated with AUDA and untreated mice. (b) Mice body weight measurement after 7 consecutive days of AUDA treatment. Body weight measurements of mice after 7 consecutive days of AUDA treatment. (c) Immunohistochemical staining (×200) depicting the expression of IL-1β, TNF-α, and IL-6 in ATs. (d) Detection of protein secretion levels for IL-1β, IL-6, and TNF-α in serum using ELISA assay (*n* = 6). (e) Assessment of inflammatory cell infiltration around the bronchioles at ×200 magnification using H&E staining. Detection of subepithelial collagen deposition through Masson’s trichrome stain, and (f) evaluation of sEH expression in mouse lung tissue through immunohistochemical staining (scale bar, 100 μm). Reproduced with permission from Ref. [97], © Elsevier Inc. 2023.

Beyond the IRF/STAT axis, peroxisome proliferator-activated receptor gamma (PPARγ) is another important regulator of M2 polarization [95]. PPARγ is activated by oxidized phospholipids and fatty acids, enhancing the expression of M2 markers including Arg1, CD206, and IL-10 [96]. The systemic inflammation driven by obesity-associated adipose tissue (AT) dysfunction often extends beyond metabolic dysregulation, contributing to comorbid conditions such as asthma. This crosstalk is mediated by shared inflammatory pathways, including the recruitment and polarization of MΦs [97]. A mouse model of obese asthma, induced through a combination of high-fat diet (HFD) feeding and ovalbumin (OVA) sensitization, was utilized to assess the impact of AUDA on parameters such as airway inflammation, airway hyperresponsiveness (AHR), and pulmonary pathological alterations. To determine the potential impact of 12-(3-adamantan-1-ylureido)dodecanoic acid (AUDA) pretreatment on body weight in obese asthma, the weight of mice in each group was recorded. As depicted in Figs. 3(a) and 3(b), the DIO-OVA group (HFD + OVA) showed a significant increase in weight (*p* < 0.01) compared to control mice (normal chow diet). Nonetheless, pre-treatment with AUDA, even at a dosage of 30 mg/kg (*p* = 0.25), did not result in a noteworthy decrease in mouse weight. This could be linked to the relatively brief duration of administration. Moreover, immunohistochemistry was employed to evaluate the expression of inflammation-related factors, namely IL-1β, TNF-α, and IL-6 in ATs (Fig. 3(c)). They verified that AUDA reduced the expression of pro-inflammatory factors, such as IL-1β, IL-6, and TNF-α, in ATs and serum. As illustrated in Fig. 3(d), the OVA group (normal chow diet + OVA) and DIO-OVA group exhibited substantial elevations in serum TNF-α, IL-1β, and IL-6 levels. Notably, these increases were markedly attenuated in the AUDA treatment group, with the most significant reduction observed in the 30 mg/kg AUDA group (HFD + OVA + AUDA 30 mg/kg). Subsequently, H&E staining was utilized to evaluate the infiltration of inflammatory cells in lung tissue. Figure 3(e) shows a noteworthy increase in inflammatory infiltration around the airway and peripulmonary vessels in the OVA, DIO, and DIO-OVA groups compared to the control group. However, mice pretreated with AUDA at doses of 3, 10, or 30 mg/kg exhibited a dose-dependent reduction in inflammatory cell infiltration. Figure 3(f) illustrates a substantial increase in sEH expression in the DIO (HFD), OVA, and DIO-OVA groups. However, pre-treatment with AUDA demonstrated a dose-dependent down-regulation of sEH expression. Their conclusion highlighted the efficacy of AUDA in diminishing inflammation within ATs, leading to a subsequent reduction in systemic inflammation.

The role of ATs fibrosis has been recognized as a novel factor contributing to the pathomechanism of metabolic disorders associated with obesity. The morphological alterations in cells ultimately lead to fibrosis and dysfunction in ATs. Therefore, preventing WATs fibrosis is useful for enhancing systemic energy and glucose balance. With its anti-obesity effects, Zhang et al. discovered that sulforaphane (SFN) markedly reduced WAT fibrosis and improved insulin resistance in diet-induced obesity mouse models [98]. According to their findings, SFN decreased inflammation and encouraged the polarization of MΦs towards M2 in ATs, offering protection against fibrosis.

## 3 NP and nanomedicine therapies for modulating MΦ polarization in obesity

Nanomedicine and NPs have emerged as promising approaches for obesity treatment, offering unique strategies for immune system modulation. These systems provide opportunities to influence immune responses associated with obesity. Through targeted drug delivery and immune modulation, they offer the potential to improve insulin sensitivity, reduce chronic inflammation, and promote healthy AT function. This multidimensional approach holds promise for developing effective and personalized therapies for obesity treatment.

### 3.1 MΦ polarization

NPs can also influence MΦ polarization, which plays a crucial role in AT inflammation and metabolism. For instance, sodium salicylate-loaded NPs have been shown to induce the browning of white adipocytes via M2 MΦ polarization, offering a promising therapeutic strategy for obesity [99, 100]. A recent study by Choi et al. unveiled a novel mechanism by which sodium salicylate NPs can induce therapeutic browning of WATs through MΦ reprogramming [101]. Upon systemic administration in obese mice, the sodium salicylate NPs preferentially accumulated in adipose depots and polarized resident MΦs towards an anti-inflammatory M2 phenotype. This M2 polarization was mediated by the upregulation of heme oxygenase-1 (HO-1), a key regulator of the antioxidant response. The M2 MΦs subsequently secreted browning factors like norepinephrine and meteorin-like protein, which stimulated the browning program in adjacent white adipocytes, leading to the formation of metabolically active beige fat cells. Remarkably, this NP-induced adipose browning resulted in significant improvements in whole-body metabolism, including enhanced energy expenditure, reduced adiposity, better glucose tolerance, and improved insulin sensitivity. This study highlights the therapeutic potential of NP-mediated MΦ reprogramming as a novel strategy for inducing the browning of WAT to combat obesity and metabolic disorders.

**Figure 4:**
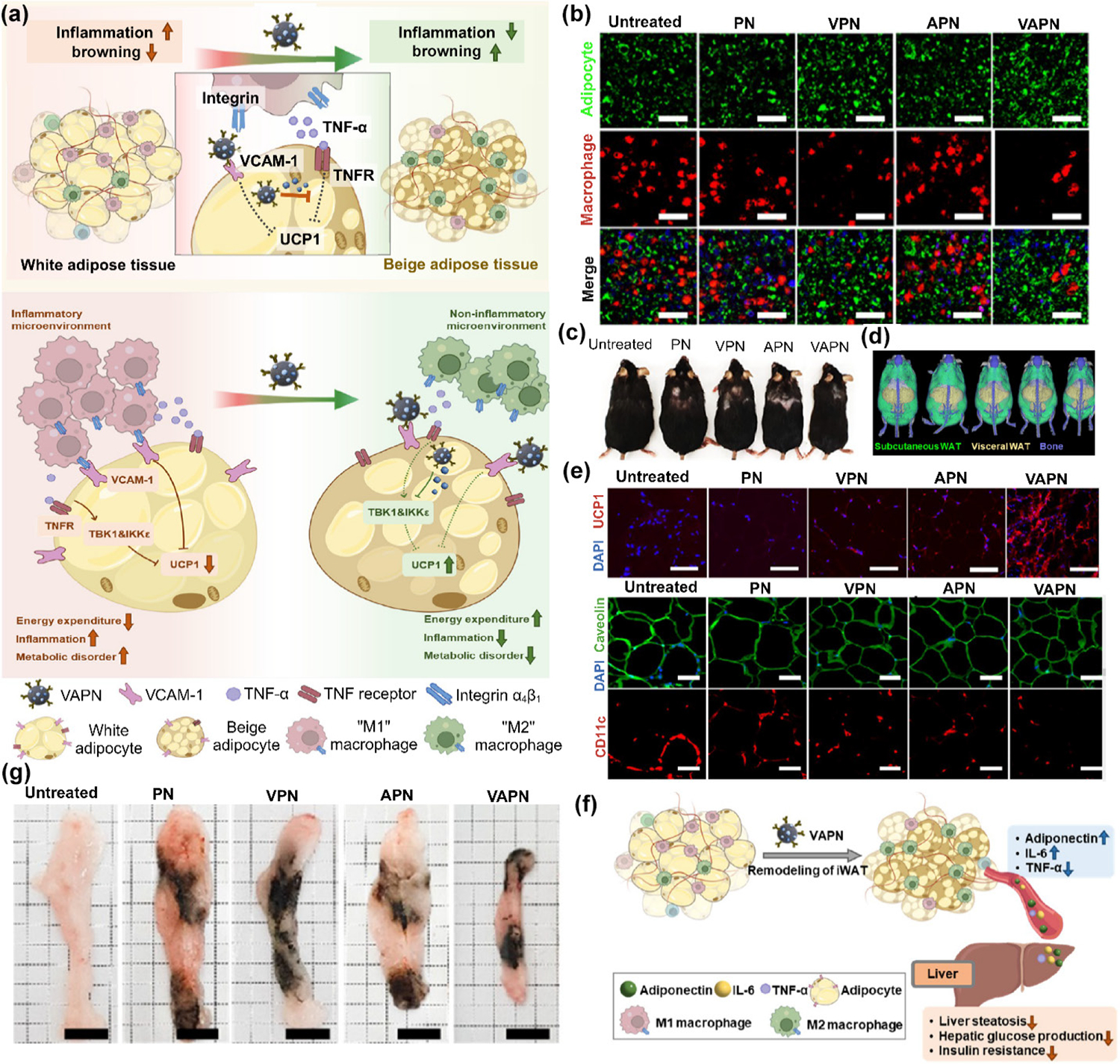
Nanomodulator-mediated remodeling of AT immune microenvironment for obesity treatment. (a) Proposed mechanisms of function for VAPN in anti-obesity treatment. VAPN blocks VCAM-1 on adipocyte surfaces, reducing direct interaction with MΦs. Amlexanox counters the repressing impacts of inflammatory cytokines on adipocyte browning. Additionally, VAPN enhances energy expenditure and remodels inflammatory microenvironments in ATs, supporting its anti-obesity outcomes. (b) MΦ adhesion to NP-bound ATs. Following co-incubation of MΦs with NP-treated adipocytes, MΦ adhesion to adipocytes was visualized using confocal microscopy (isolated adipocytes were labeled with BODIPY, and MΦs were stained with CellTracker Red CMTPX dye). Scale bar: 50 μm. (c) Actual photos of mice following 4 weeks of treatment with different NPs. (d) Micro-CT images of ATs were collected from the mice treated with different conditions. (e) Immunohistochemistry visualization of UCP1 expression (red) and CD11c+ MΦs (red) in iWAT. Scale bar: 50 and 100 μm. (f) Illustration of the proposed mechanism for the VAPN systemic therapeutic effect. (g) Actual photographs of iWAT collected from different treated groups. Scale bar: 1 cm. Reproduced with permission from Ref. [102], © American Chemical Society 2024.

In obesity, the interactions between adipocytes in WATs and pro-inflammatory M1 MΦs play a crucial role in propagating inflammation and metabolic dysfunction [102]. By modulating the immune cell composition and cytokine profile in ATs, the nanomodulators help create an anti-inflammatory microenvironment that is less conducive to obesity development and associated with metabolic complications. The nanomodulators discussed in a study by Li et al. reshaped the AT immune microenvironment to counteract obesity [102]. The nanomodulators, designed as vascular cell adhesion molecule (VCAM)-1 antibody-conjugated and amlexanox-loaded polydopamine nanoparticles (VAPN), aim to modulate the crosstalk between adipocytes and MΦs in ATs (Fig. 4(a)). The adhesion of MΦs to adipocytes treated with various methods was examined using confocal microscopy (Fig. 4(b)), revealing fewer MΦs adhering to adipocytes treated with VPN or VAPN. VAPN showed greater binding to VCAM-1-expressing adipocytes and reduced the interaction of adipocytes with MΦs compared to NPs lacking antibody modification or amlexanox. Additionally, in diet-induced obese mice, repeated subcutaneous administration of VAPN notably reduced body weight (Fig. 4(c)) and significantly increased the populations of beige adipocytes as well as ameliorated inflammation in WATs. Subsequent Micro-CT imaging demonstrated that the VAPN-treated group exhibited the lowest fat content (Fig. 4(d)). As depicted in Fig. 4(e), the enhanced UCP1 expression (in red) in iWAT was further assessed via fluorescence microscopy. Additionally, the presence of CD11c+ MΦs in iWAT showed a two-fold reduction in mice treated with VAPN compared to APN, as illustrated in Fig. 4(f). The localized application of VAPN exerted systemic metabolic effects, reducing metabolic disorders like insulin resistance and liver steatosis. Figure 4(g) displays actual photographs of iWAT, confirming a significant reduction in the fat depot mass in VAPN-treated mice compared to APN-treated mice. This study suggests that nanomodulator-mediated restructuring of the AT immune microenvironment has the potential to treat obesity by modulating the interactions between adipocytes and immune cells, particularly MΦs. This approach aims to create an anti-inflammatory milieu in ATs to counteract the pro-inflammatory state associated with obesity and its metabolic complications [102].

**Figure 5:**
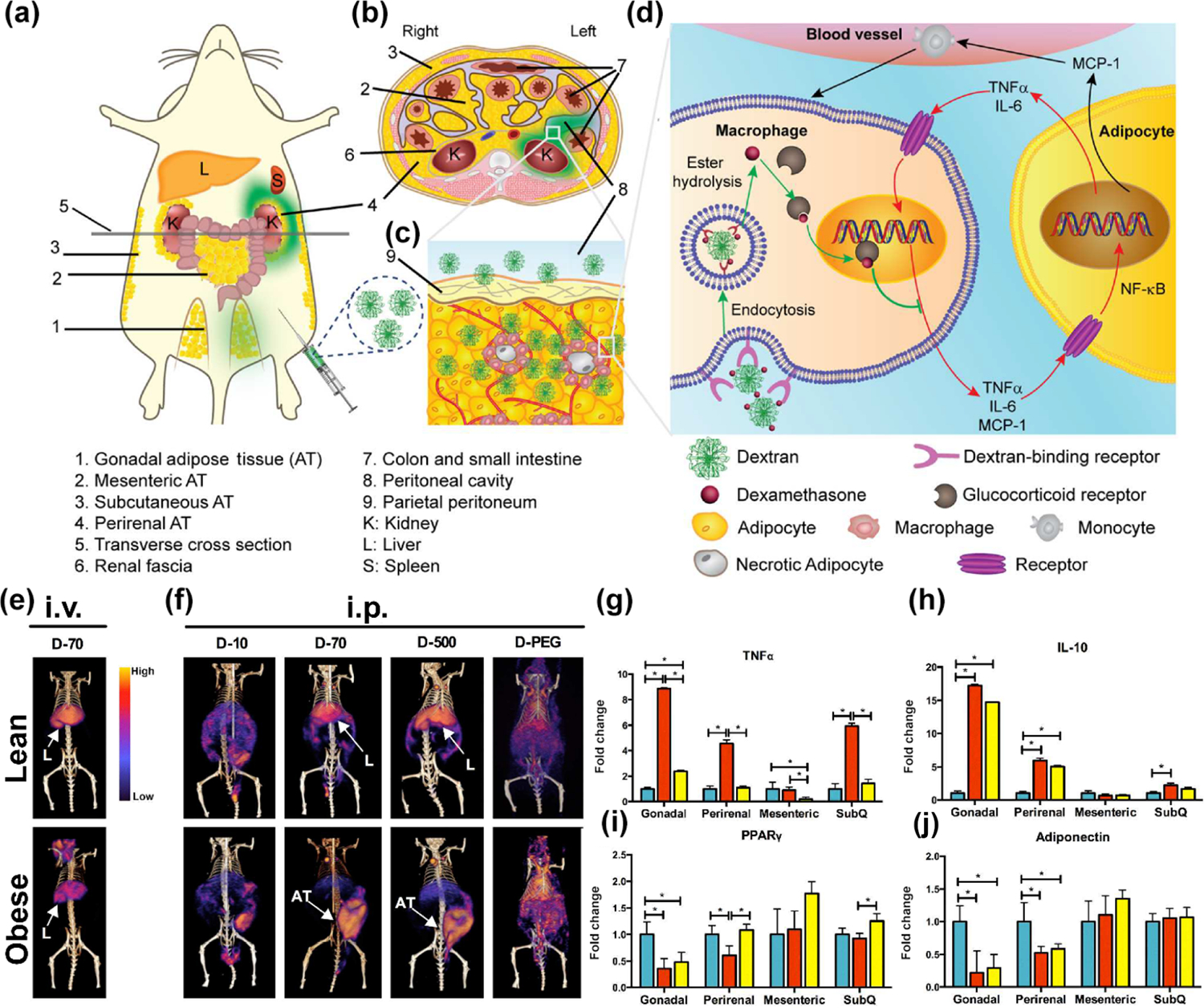
Nanoscale polysaccharides based on biocompatible dextran. (a) Dextran-dexamethasone conjugate mechanism in obese VAT: MΦ uptake and uncoupling of the paracrine interaction between M1 and adipocytes. (a) Accumulation of dextran conjugates (green) in left perirenal and gonadal ATs after intraperitoneal (i.p.) injection. (b) Transverse abdominal cross-section showing dextran solution (green) distribution. (c) The dextran conjugates rapidly associate with M1 MΦs in inflamed AT due to their transport across the peritoneum, allowing direct access to interstitial cells. (d) Disruption of the inflammatory paracrine loop between M1 and adipocytes with conjugates. In obesity, hypertrophied adipocytes produce MCP-1, recruiting monocytes and promoting M1 differentiation. M1 releases pro-inflammatory cytokines (TNFα and IL-6), enhancing adipocyte inflammation via NF-κB activation. Dextran-dexamethasone conjugates are endocytosed by M1, releasing dexamethasone intracellularly. Dexamethasone binds to glucocorticoid receptors, inhibiting pro-inflammatory gene transcription and attenuating the inflammatory cycle. (e) and (f) Biodistribution of dextran nano-carriers in lean versus obese mice. (e) Reconstructed PET/CT images comparing the biodistribution of radiolabeled dextran nanocarriers (D-70-rad) in lean and obese mice. The images were acquired 24 h after jugular vein injection. (Top row) Lean mice showing the distribution pattern of D-70-rad. (Bottom row) Obese mice demonstrate altered biodistribution of D-70-rad, with notable accumulation in AT depots. (f) PET/CT images showing the top row: lean and the bottom row: obese mice 24 h after i.p. injection with conjugates. (g)–(j) *In vivo* anti-inflammatory impacts of D-70-drug in VAT of the obese mouse. Relative mRNA expression levels of genes in four adipose depots are compared among lean, obese, and obese mice treated with D-70-drug. L: Liver, dextran molecular weights of 10 kDa (D-10), 70 kDa (D-70), and 500 kDa (D-500). Reproduced with permission from Ref. [104], © American Chemical Society 2016.

NF-κB regulates gene expression in MΦs by influencing gene transcription, thereby promoting inflammation [103]. Preventing NF-κB expression can be positive in treating obesity and obesity-related metabolic disorders. Tao et al. presented an innovative nanotechnology-based approach to obesity treatment [104]. The researchers developed nanoscale polysaccharides based on biocompatible glucose polymers (dextran) designed to efficiently target ATMs in obese mice and prevent the paracrine loop between M1 and adipocytes and adipocytes (Fig. 5(a)). Upon entering M1, the dextran conjugates release dexamethasone, which then binds to glucocorticoid receptors. This binding inhibits the expression of NF-κB and suppresses the transcription of pro-inflammatory genes. Due to their proximity to the injection site in the lower left ventral region, the perirenal and gonadal fat pads are likely to come into direct physical contact with the administered fluid (Fig. 5(b)). PET/CT imaging showed that the delivered dose of dextran was uniformly distributed within this AT, indicating that once perfused, it evenly accessed all regions of the tissue (Fig. 5(c)). In the context of obesity-induced inflammation, it is crucial to recognize that both MΦs and adipocytes in visceral adipose tissue (VAT) contribute to the expression of pro-inflammatory genes. These cell types participate in a feed-forward paracrine loop, as illustrated in Fig. 5(d). This interplay makes it challenging to precisely determine the extent to which each cell type is affected at the gene expression level when evaluating the effects of targeted interventions. This complexity underscores the importance of considering broader cellular interactions within ATs when developing targeted therapies for obesity-related inflammation. To study the distribution of dextran nano-carriers in lean and obese mice, radiolabeled conjugates were injected into age-matched C57BL/6J mice that were fed either a low-fat diet or an HFD. Figure 5(e) displays PET/CT images showing that 24 h after D-70 (molecular weight of dextran is 70 kDa (D-70)) was administered via jugular vein injection, it mostly accumulated in the liver for both lean and obese mice. Conversely, as illustrated in Fig. 5(b), a significantly different pattern emerged after i.p. administration, with a substantial portion of D-70 and D-500 accumulating in the obese mice VAT. This effect was not seen in lean animals, where the liver continued to be the main uptake site. Additionally, in comparison to lean mice, obese mice demonstrated elevated levels of pro-inflammatory markers (TNFα), increased expression of the anti-inflammatory cytokine IL-10, decreased levels of regulators of adipogenesis, lipid metabolism, and glucose homeostasis (such as adiponectin and PPARγ), and higher expression of leptin (Figs. 5(g)–5(j)). By specifically targeting ATMs, which play a crucial role in obesity-related inflammation, this method offers the potential for more precise treatment of obesity-induced inflammation and its associated metabolic complications while potentially minimizing systemic side effects associated with traditional drug delivery methods. This work underscores the promise of using targeted NP systems to address the underlying inflammatory processes in obesity, opening new avenues for therapeutic interventions in this complex metabolic disorder.

**Figure 6:**
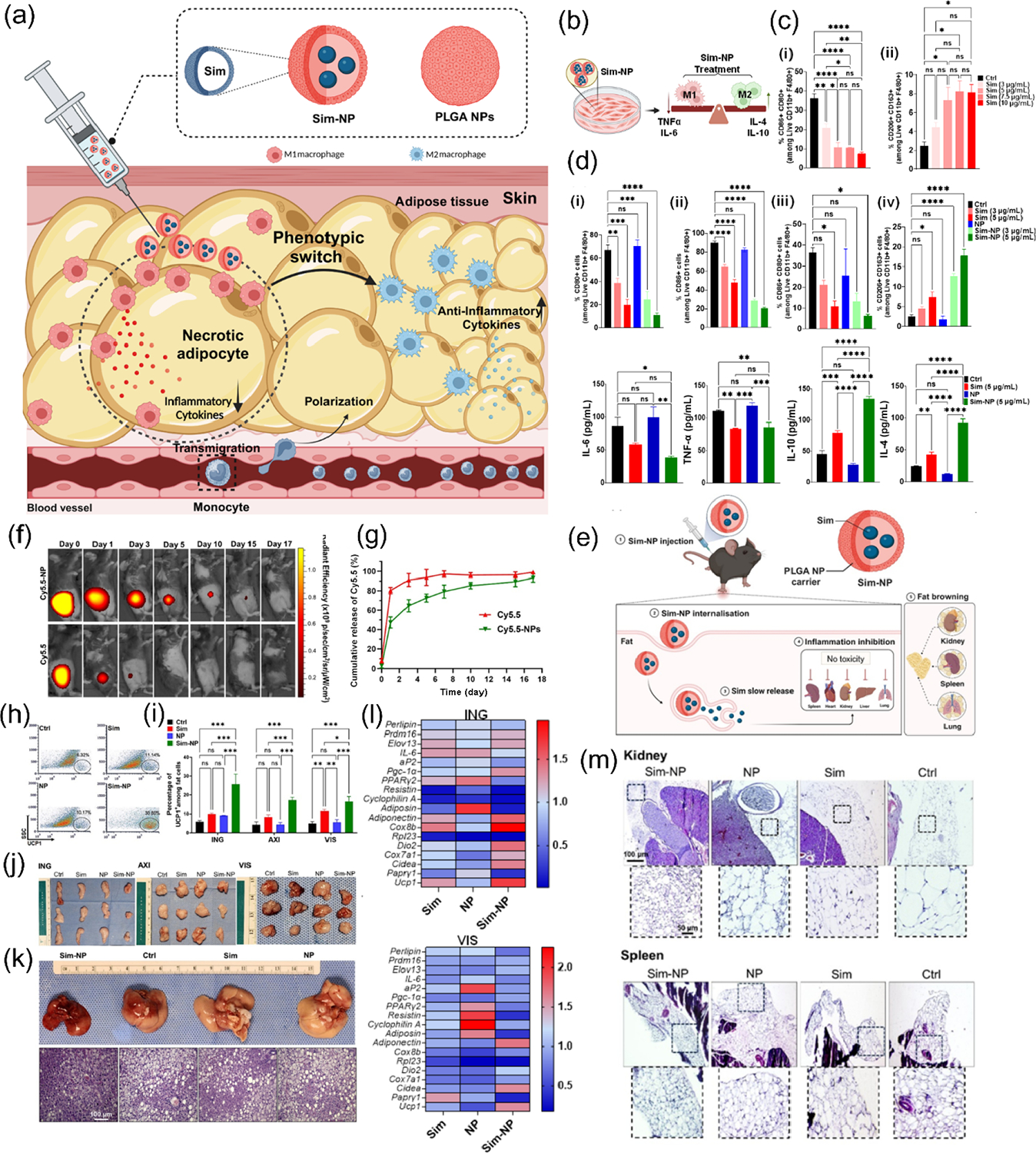
Effects of Sim-loaded NPs on AT browning and obesity-related parameters in high-fat diet mice. (a) The schematic diagram illustrates the design and anticipated effects of Sim-NPs in obesity treatment, showcasing their gradual release mechanism to modulate MΦ polarization from M1 to M2, promote white-to-brown fat conversion, and reduce inflammation in WAT by adjusting adipose function and cytokine secretion. (b)–(d) The polarization profile of BMDMs treated with Sim-NPs demonstrates the shift from pro-inflammatory M1 to an anti-inflammatory M2 phenotype. This modulation highlights the potential of Sim-NPs to reduce inflammation and promote obesity treatment strategies. (e) *In vivo* evaluation of Sim-NPs in obesity treatment. (f) Biodistribution and localized retention of fluorescent NPs in ATs. Fluorescence imaging of Cy5.5 or Cy5.5-NPs distribution in ING WAT. (g) Quantification of NP retention and distribution in ING WAT of injected mice, illustrating the potential for long-term therapeutic delivery in obesity treatment. Multifaceted effects of Sim-NPs on ATs and livers in obese mice. (h) Flow cytometry scatterplot showing increased UCP1-positive fat cells in the Sim-NP treated group. (i) Quantification of UCP1 marker in ING, AXI, and VIS AT by flow cytometry. (j) Visual comparison of ING, AXI, and VIS AT after 4-week treatment (*n* = 10 per group, three representative animals shown). (k) Liver weight comparison (upper panel) and H&E stained liver sections (lower panel), demonstrating Sim-NP impact on hepatic health. (l) Gene expression profile of ATs and liver following Sim-NP treatment. (m) The image demonstrates the safety profile and efficacy of Sim-NPs in reducing fat droplets across various AT depots, highlighting the potential of this approach for systemic fat reduction in multiple anatomical locations. Reproduced with permission from Ref. [105], © American Chemical Society 2024.

Targeting inflammatory MΦs for local AT browning presents a new immunoengineering approach for obesity treatment. Recent research has unveiled a promising approach to obesity treatment using simvastatin-loaded polymeric NPs (Sim-NPs) to target inflammatory MΦs in ATs (Fig. 6(a)). This innovative strategy addresses the chronic low-grade inflammation associated with obesity [105]. Our team’s study demonstrated that Sim-NPs effectively inhibit obesity-related inflammation, control white fat production, and enhance ATs modulation. In both *in vitro* experiments and a mouse model of diet-induced obesity, Sim-NPs exhibited potent anti-inflammatory effects, improved MΦ polarization modulation, and induced AT browning. The polarization profile of BMDMs using Sim-NPs demonstrates their ability to modulate MΦ polarization from the pro-inflammatory M1 phenotype to the anti-inflammatory M2 phenotype (Figs. 6(b)–6(d)). This shift in MΦ polarization indicates the potential of Sim and Sim-NPs to reduce inflammation and promote tissue repair in obesity treatment strategies. The visual distribution of Cy5.5-conjugated poly(lactide-co-glycolide) (PLGA) NPs in mice inguinal white adipose tissue (ING WAT) demonstrates localized retention and distribution over time (Figs. 6(f) and 6(g)). Quantitative analysis of the injected mice (*n* = 10) reveals that these NPs exhibit sustained presence in the ING WAT, suggesting their potential for prolonged local drug delivery and therapeutic effects in ATs. This reduction in fat accumulation was observed across different anatomical locations, indicating the potential of Sim-NPs as a systemic approach to combat obesity-related fat deposition in multiple body regions. Additionally, Sim-NPs demonstrated significant WAT browning effects in HFD mice, showcasing their potential to modulate the immune system, alter MΦ polarization, and treat obesity. The treatment resulted in increased UCP1-positive cells (Figs. 6(h) and 6(i)), reduced lipid droplet area and size in various AT depots, visibly smaller AT volumes (Fig. 6(j)), and improved liver health (Fig. 6(k)), highlighting the systemic benefits of this targeted NP-based approach in obesity management. As seen in Fig. 6(l), quantitative PCR analysis revealed significant changes in brown and white adipocyte gene expressions across multiple AT depots (ING, visceral (VIS), testis (TES), axillary (AXI), and thyroid (TYR)) and liver in response to Sim-NP treatment, providing evidence for the browning effect and metabolic improvements observed in the obesity model. *In vivo*, assessments of Sim-NPs demonstrated a favorable safety profile and significant efficacy in reducing fat droplets in ATs associated with various organs and tissues (Figs. 6(e) and 6(m)). The success of this NP-based therapy highlights the potential of immunoengineering strategies in obesity treatment. By specifically modulating ATMs, which play a crucial role in ATs inflammation and obesity-related metabolic diseases, this approach opens new avenues for developing more effective obesity treatments. As obesity continues to pose significant public health challenges worldwide, innovative solutions like Sim-NPs represent a step forward in addressing this complex issue through localized immunomodulation.

**Figure 7:**
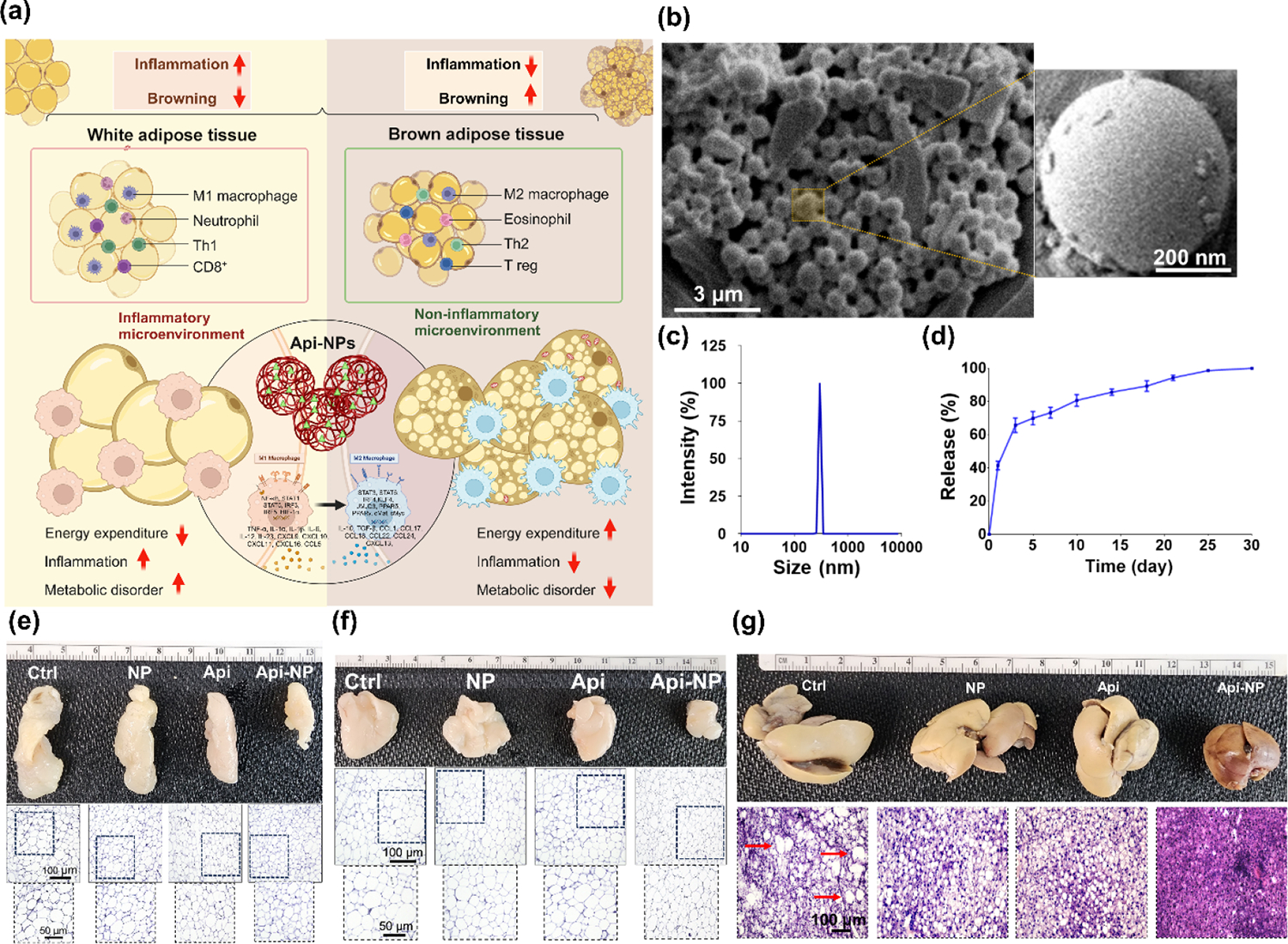
The impact of Api-NPs on inflammation, adipose tissue browning, and liver health in obesity management. (a) Schematic representation of Api-NPs’ design and their anticipated effects on obesity-related inflammation and AT browning. Api-NPs facilitate the sustained release of Api, specifically targeting ATMs to shift polarization from pro-inflammatory M1 to anti-inflammatory M2. This immunomodulation reduces inflammation and pro-inflammatory cytokine secretion while creating a microenvironment that promotes WAT browning. These combined effects contribute to improved adipose function, reduced inflammation, and the mitigation of obesity-associated metabolic dysfunctions. (b) SEM images reveal the spherical morphology and uniform size distribution of Api-NPs, crucial for controlled drug release. Scale bar: 3 μm; inset scale bar: 200 nm. (c) DLS analysis at pH 7.4 and 37 °C indicates a stable size distribution, supporting *in vivo* stability. (d) The *in vitro* release profile of Api from Api-NPs in mouse serum at 37 °C over 30 days demonstrates sustained drug release. With an encapsulation efficiency of approximately 73.2% ± 4.3%, Api-NPs ensure effective delivery, prolonged circulation, and optimized immunomodulatory effects on adipose tissue. (e) and (f) Api-NPs induce WAT browning in HFD-fed mice by influencing immune modulation and MΦ polarization. Representative images and H&E staining of (e) inguinal WAT and (f) epididymal WAT after 4 weeks of treatment. Images from three representative mice per group illustrate structural changes in adipose tissue. (g) Excised livers and H&E-stained sections show the effects of Api-NP treatment. Livers from untreated HFD-fed mice appeared enlarged and pale, indicative of hepatic steatosis, while those from Api-NP-treated mice were smaller and healthier in color, suggesting a protective effect against fatty liver disease. Reproduced with permission from Ref. [106], © Elsevier B.V. 2025.

In a recent study by Mohaghegh et al., the effects of PLGA NPs loaded with apigenin (Api-NPs) were developed to modulate MΦ polarization in AT and promote adipocyte browning in a HFD-induced obesity mouse model [106]. The study demonstrated that Api-NPs effectively shifted MΦ polarization from the pro-inflammatory M1 phenotype to the anti-inflammatory M2 phenotype, leading to a significant reduction in inflammatory markers, adipocyte size, and metabolic improvements in ATs (Fig. 7(a)). Physicochemical characterization of Api-NPs revealed a uniform spherical morphology, as confirmed by scanning electron microscopy (SEM), with a homogeneous size distribution (Fig. 7(b)). Dynamic light scattering (DLS) analysis further supported these findings, indicating favorable properties for prolonged systemic circulation and efficient AT targeting. Importantly, high-performance liquid chromatography (HPLC) analysis demonstrated a high encapsulation efficiency of Api within PLGA NPs. The controlled release profile of Api-NPs, evaluated under physiological conditions, indicated sustained drug release over time, ensuring prolonged therapeutic effects (Figs. 7(c) and 7(d)). *In vivo* administration of Api-NPs in HFD-induced obese mice led to a marked decrease in body weight and AT mass. Histological analyses of AT revealed significantly smaller adipocytes in the Api-NP-treated group compared to untreated controls, supporting the hypothesis that MΦ modulation can create a microenvironment conducive to adipocyte browning (Figs. 7(e) and 7(f)). Furthermore, examination of liver tissue from treated mice showed a noticeable reduction in hepatic steatosis, with livers exhibiting a healthier reddish-brown color compared to the enlarged, pale livers observed in untreated obese mice (Fig. 7(g)). Overall, these findings highlight the potential of Api-NPs as a promising therapeutic strategy for obesity management. The combination of immunomodulatory effects, adipocyte browning induction, and sustained localized drug delivery positions Api-NPs as a viable approach for addressing obesity-related inflammation and metabolic dysfunction.

### 3.2 Browning of WAT

Recent advances in nanomedicine have led to the development of various NPs, such as PLGA, poly(ethylene glycol), polyethylenimine, lipid NPs (LNPs), and hepatitis B core protein virus-like particles (VLPs), for the targeted delivery of browning agents to WATs. These NPs can enhance efficiency and reduce the side effects of browning agents by ensuring tissue-specific delivery [107]. According to Decuzzi et al., spherical NPs with an average diameter of 200 nm have been developed for MΦ-selective delivery of rosiglitazone (Rosi) as a potent browning inducer in subcutaneous WATs [108]. They used PLGA and PVA polymers as constituents due to their biodegradability and biocompatibility properties. Consequently, this nano platform offered a novel method to deliver agonists for nuclear receptor ligands, like PPARγ, into monocytes and MΦs to attenuate chronic disease states mediated by MΦ inflammation. PPARγ has been identified as effective in attenuating MΦ inflammatory responses implicated in obesity metabolic complications and atherosclerosis. Based on their findings, Rosi-NPs induced PPARγ target genes with the same, or even higher, levels than free Rosi with bone marrow-derived macrophages (BMDMs). Furthermore, HFD-fed LDLR−/− mice were intravascularly injected with Rosi-NPs, which reduced MΦ inflammation in both the WAT and liver. This effect was observed without any impact on genes associated with lipid metabolism or cardiac function, which are known to be affected by systemic Rosi delivery. The selective delivery of Rosi to MΦ thus represents a promising approach for mitigating inflammation without exposing the body to the known side effects of systemic Rosi exposure. In the other study using Rosi, Hiradati et al. developed locally administered Rosi-loaded NPs for the precise activation of PPARγ in adipocytes, resulting in the browning of WATs [109]. The dual-targeted Rosi-NPs were modified by incorporating a designated peptide to target prohibition expressed in adipocytes. Additionally, a cell-penetrating peptide has been incorporated to enhance cellular uptake and regulate intracellular trafficking. The prohibition-targeted nanoparticles (PTNP) system was formulated by utilizing two distinct forms of PEG spacers, namely PEG2kDa and PEG5kDa. The latter tips were modified with the prohibition targeting peptide sequence (CKGGRAKDC). The R8-modified PTNP (R8-PTNP) is a composite comprising PTNP modified with R8. This peptide functions as a cell-penetrating agent to further enhance cellular uptake while regulating intracellular trafficking. The research results suggest that Rosi-NPs, modified with a single ligand, were internalized into mature adipocytes and induced browning activity *in vitro*. However, their impact on the body weight of the diet-induced obese mice model was not significant. The dual-targeted Rosi-NPs exhibited significant browning activity, both *in vitro* and *in vivo*, and effectively arrested the progression of obesity, as confirmed by the reduction in the size of hypertrophied adipocytes, while not inducing any discernible systemic adverse effects. The novelty of the suggested NPs can be attributed to the utilization of two ligands, specifically a peptide that selectively targets inhibition and the R8 peptide. It was only the dual-ligand system that elevated the levels of UCP1 in AT and substantially suppressed body weight gain.

In another study, Lin et al. reported the novel aptamer-functionalized nano gel of gold nanoclusters (AuNCs) for a targeted delivery vehicle of docosahexaenoic acid (DHA) [110]. The MA-33 aptamer has a sequence of 5’-GTT ACC GCG GTG AAG GGT GGA TGT GTC TGG ACG CTA TAT C-3’. The human body requires DHA, a crucial nutrient belonging to the ω-3 unsaturated fatty acid family. Moreover, recent research has revealed that DHA could efficiently trigger the browning process in WAT. Following DHA treatment, the adipocytes exhibited distinctive features associated with browning, such as elevated levels of UCP1 and mitochondria, increased glucose consumption, and reduced triglyceride levels [111]. This observation suggests that DHA may have significant implications for regulating adipocyte metabolism. Unfortunately, when using DHA as a browning agent, numerous difficulties arise.

**Figure 8:**
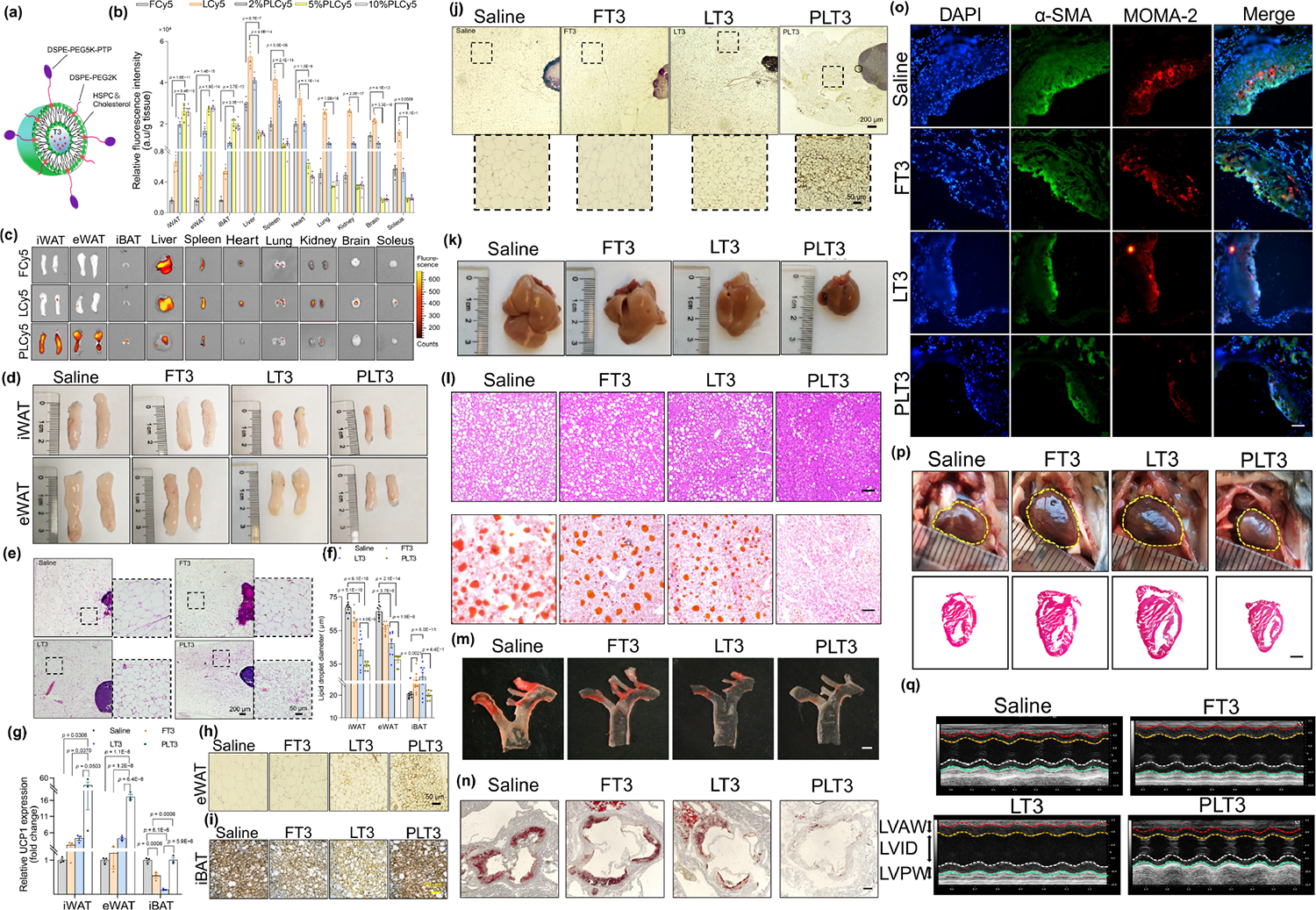
Schematic, fluorescence distribution, and therapeutic evaluation of PLT3 in obese mice. (a) A schematic representation of the structure of triiodothyronine (T3)-encapsulated PTP-modified liposomes (PLT3), which consist of HSPC, cholesterol, DSPE-PEG2K, DSPE-PEG5K-PTP, and T3. The present study involves constructing and assessing liposomal NPs that allow for adipose-selective drug delivery to track the distribution of liposomes *in vivo*; Cy5 was encapsulated within them. Additionally, 8-week-old male C57BL/6N mice were intraperitoneally (IP) injected with free Cy5 (FCy5), LCy5, or PLCy5s. Mice were sacrificed 8 h after injection. (b) The relative fluorescence intensities in various adipose tissues such as iWAT, eWAT, and iBAT, as well as in organs such as the liver, spleen, heart, lung, kidney, brain, and soleus. These intensities were determined with a fluorimeter. (c) The analysis of *ex vivo* fluorescence imaging for various tissues. (d) The gross appearance of both iWAT and eWAT. (e) H&E staining of iWAT, eWAT, and iBAT. (f) The diameter of lipid droplets in iWAT, eWAT, and iBAT. Further information on UCP1 protein expression was obtained through immunohistochemistry (IHC) staining, as illustrated in (g)–(j) WAT, iBAT, and iWAT, respectively. Obese mice that were induced with HFD were subjected to various forms of T3 or saline for 32 days. (k) Representative depictions of the macroscopic characteristics and (l) histological staining with H&E (upper panel) and Oil Red O (lower panel) of liver tissue specimens, with accompanying scale bars of 100 μm (H&E staining) and 50 μm (Oil Red O staining). (m) Representative photographs depicting aortas in the en face preparation, which were subsequently stained with Oil Red O, were obtained and analyzed. The scale bar utilized to measure the images was set at 1 mm. (n) Representative histological images of the aortic sinus, which were also stained with oil red O, were acquired and examined. The scale bar employed to measure these images was set at 100 μm. (o) Immunofluorescence analysis utilizing α-smooth muscle actin (α-SMA) and monocyte/MΦ-2 (MOMA-2) was conducted in the atherosclerotic lesion zone of the aortic sinus. The scale bar for this analysis was determined to be 50 μm. (p) Representative images depicting the gross appearance of the hearts (upper panel) and H&E-stained cross-sections of the hearts (lower panel) were obtained. The scale bar for this analysis was determined to be 2 mm. (q) Representative M-mode echocardiographic images of the left ventricular (LV) short-axis were also captured. The LV anterior wall thickness was measured as LVAW, the LV inner dimension was measured as LVID, and the LV posterior wall thickness was measured as LVPW. Reproduced with permission from Ref. [112], © Chen, K. et al. 2022.

Thyroid hormone (TH) has the potential to mitigate obesity as a potent thermogenic agent; however, the systemic administration of TH has limited clinical significance in terms of weight reduction [112]. Xu et al. achieved adipose-selective drug delivery by encapsulating triiodothyronine (T3) in liposomes modified with an adipose-homing peptide (PTP) [112]. The encapsulation of T3 into liposomal NPs (PLT3) has been demonstrated in Fig. 8(a). The liposomal NPs were further modified with an adipose-homing peptide, PTP, which possesses the amino acid sequence of CKGGRAKDC, resulting in the formation of PLT. To confirm the selective targeting ability of PTP-modified liposomal NPs towards ATs and ascertain the most suitable PTP concentration in liposomes and administration route, diverse concentrations of Cy5-encapsulated liposomes modified with varying molar ratios of PTP (PLCy5s) were employed to encapsulate the fluorescent dye Cy5. The results indicate that mice receiving 5% PLCy5 demonstrated a significant increase in the accumulation of Cy5 in their ATs, while other non-ATs exhibited significantly lower levels of Cy5 compared to Cy5-encapsulated unmodified liposomes (LCy5) and 2% PLCy5 (Figs. 8(b) and 8(c)). Further analysis of specific adipose depots revealed a significant reduction in the quantity of inguinal subcutaneous white adipose tissue (iWAT) and epididymal white adipose tissue (eWAT) in mice treated with PLT3 compared to those in the FT3 and LT3 treatment groups (Fig. 8(d)). Similarly, their findings indicate that the dimensions of adipocytes in iWAT and eWAT of mice subjected to PLT3 treatment were significantly reduced compared to the other T3-treated groups (Figs. 8(e) and 8(f)). This suggests that PLT3 is a more potent agent in mitigating lipid accumulation in white adipocytes. Furthermore, the findings indicated that the PLT3 exhibited considerably higher efficacy in stimulating the transcriptional activity of multiple browning markers and in elevating the abundance of UCP1 protein (Fig. 8(g)), as well as the occurrence of multilocular UCP1+ adipocytes, both in the iWAT (Fig. 8(j)) and eWAT (Fig. 8(h)).

Figures 8(g) and 8(i) demonstrate that the interscapular brown adipose tissue (iBAT) of mice subjected to PLT3 treatment showed a minor rise or maintained their initial levels of mRNA concentrations of several browning indicators, the extent of UCP1 protein, and the multilocular UCP1+ adipocytes in the iBAT. While the size of the livers exhibited a modest decrease in mice treated with FT3 and LT3, the magnitude of reduction in PLT3-treated mice was significantly more pronounced than in the other two T3-treated groups (Fig. 8(k)). Consistently, it was observed that both H&E and Oil Red-O methods exhibited a significant degree of reduction in lipid deposition as compared to FT3 and LT3 (Fig. 8(l)). This signifies the superiority of H&E and Oil Red-O techniques in reducing lipid deposition. The efficacy of PLT3 in decreasing the dimensions of aortic atheromatous plaques was found to be considerably superior to that of LT3, in accordance with the alterations in serum lipid profiles. This was determined by utilizing Oil Red O staining of the aorta arch. Conversely, FT3 exhibited no noteworthy impact compared to the saline control (Fig. 8(m)).

Similarly, the results of histologic examination of the aortic roots, it was observed that the PLT3 cohort exhibited considerably more significant decreases in the regions of the lipid-laden atherosclerotic lesion (Fig. 8(n)), smooth muscle cell proliferation, and MΦ infiltration compared to the FT3 and LT3 groups (Fig. 8(o)). Cardiotoxicity is a prominent issue in TH pharmacotherapy. It is worth noting that despite the relatively low dose of T3 administered, both FT3 and LT3 had an observable effect on the weight and size of mouse hearts, whereas PLT3 had no effect (Fig. 8(p)). The analysis conducted through echocardiography demonstrated a significant increase in left ventricular end-diastolic anterior wall thickness (LVAW), left ventricular end-diastolic inner dimension (LVID), left ventricular end-diastolic posterior wall thickness (LVPW), left ventricle (LV) volume, LV mass, and LV ejection fraction (LVEF) due to the elevation of FT3 and LT3 (Fig. 8(q)). The administration of systemic T3 was not successful in promoting thermogenesis in both BAT and WAT. This was due to the feedback suppression of sympathetic innervation. However, the use of PLT3 therapy was able to counteract this feedback suppression on adrenergic inputs effectively. As a result, it induced browning and thermogenesis of WAT, alleviating obesity, glucose intolerance, insulin resistance, and fatty liver in obese mice. The present findings reveal WAT as a plausible target that mediates the therapeutic advantages of TH and presents a secure and effective therapeutic approach for addressing obesity and its associated complications through the administration of TH to ATs.

### 3.3 Direct anti-inflammatory and antioxidant effect

**Figure 9:**
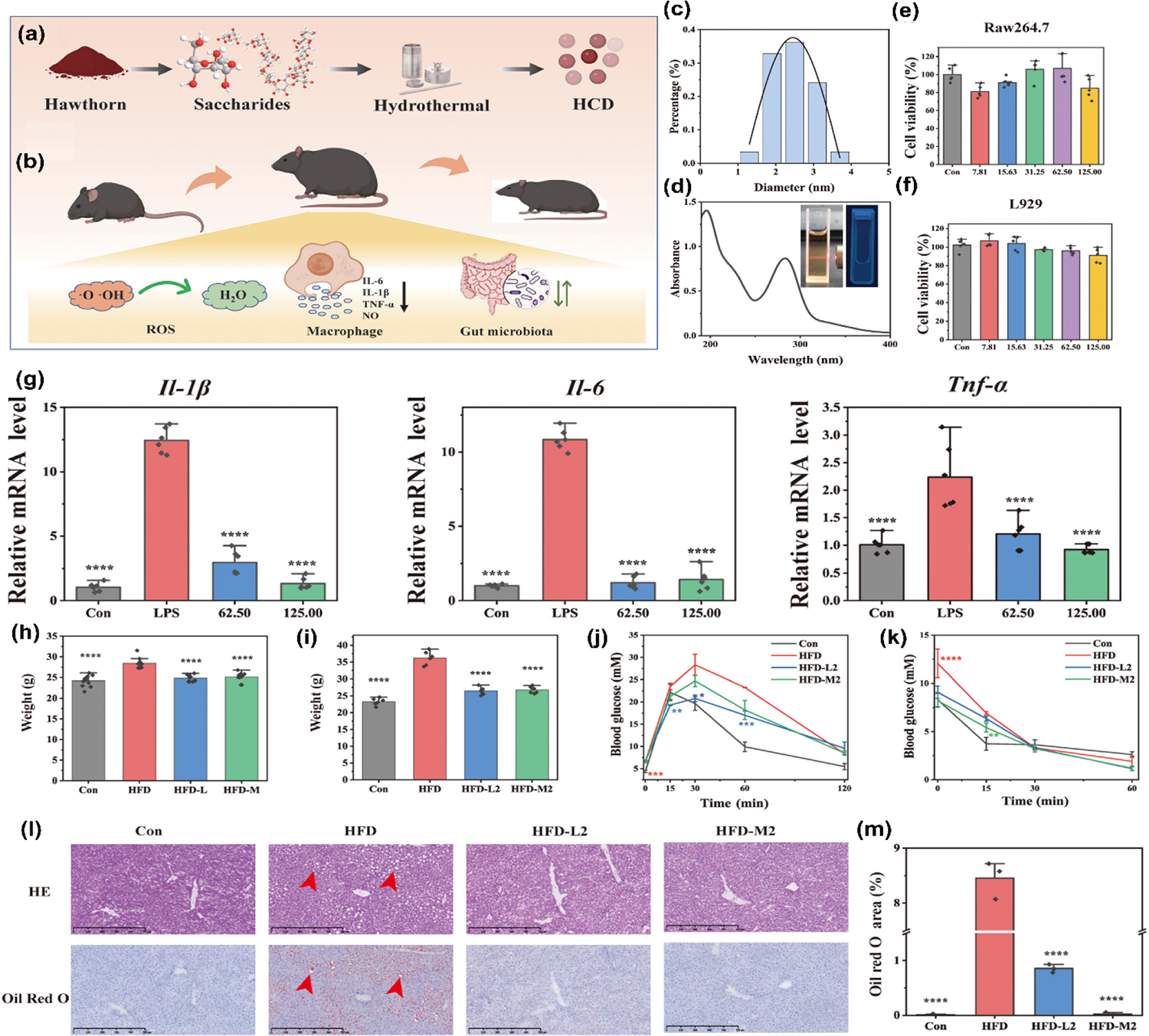
Synthesis, characterization, and therapeutic effects of HCD on obesity and inflammation in HFD mice. (a) HCD preparation and synthesis. (b) Intervention of HCD on inflammation, oxidative stress, and gut microbiome pathways in High-Fat Diet-Fed mice. (c) Particle size distribution of HCD, with an average particle size of 2.42 ± 0.53 nm. (d) UV absorption spectrum of HCD recorded in the range of 190–400 nm. The inset shows the HCD solution under daylight (brown) and under laser irradiation and UV light (blue). Effect of different concentrations of HCD on the viability of RAW264.7 (e) and L929 (f) cells. The survival rate remained above 80% at concentrations of 125 μg/mL and below, indicating the high biocompatibility of HCD. (g) Relative mRNA expression levels of inflammatory markers Il-1β, Il-6, and Tnf-α. Treatment with HCD significantly reduced the expression of these markers compared to the LPS group, indicating its anti-inflammatory effects. (h) Impact of HCD treatment on body weight. Both doses of HCD treatment significantly inhibited weight gain compared to the HFD group. (i) Effect of HCD prevention model on body weight. HCD effectively prevented weight gain in mice fed a HFD. (j) Glucose levels during the oral glucose tolerance test. (k) Glucose levels during the insulin resistance test. HCD treatment was effective in maintaining blood glucose regulation, as shown in both the glucose tolerance and insulin resistance assays. (l) H&E and Oil Red O staining of liver tissue in the HCD prevention model (*n* = 3), with red arrows indicating liver tissue vacuolization. (m) Quantitative analysis of the Oil Red O stained area in the HCD prevention model (*n* = 3). Reproduced with permission from Ref. [113], © The Royal Society of Chemistry 2025.

In a recent study, researchers developed hawthorn carbon dots (HCD), a novel therapeutic agent with promising potential in obesity treatment [113]. Synthesized through an eco-friendly hydrothermal carbonization method, HCD has demonstrated remarkable anti-inflammatory and antioxidant properties *in vitro*, addressing two critical factors in obesity pathogenesis: chronic inflammation and oxidative stress (Figs. 9(a) and 9(b)). The particle size of HCD in the nanometer range and its UV absorption spectrum were also examined, along with the color changes of the solution under different conditions (Figs. 9(c) and 9(d)). The effects of different concentrations of HCD on cell viability showed that this material has high biocompatibility (Figs. 9(e) and 9(f)). Additionally, the impact of HCD on the expression of inflammatory markers such as IL-1β, IL-6, and TNF-α highlighted its significant role in inflammation (Fig. 9(g)). *In vivo* studies using high-fat diet-induced obesity mouse models have yielded encouraging results, with HCD intervention significantly reducing body weight and hepatic lipid accumulation (Figs. 9(h) and 9(i)). Moreover, these NPs have shown the ability to enhance glucose tolerance and alleviate insulin resistance, suggesting a positive impact on metabolic health (Figs. 9(j) and 9(k)). In addition, HCD can alleviate hepatic tissue vacuolization and lipid deposition caused by HFD, as shown in Figs. 9(l) and 9(m). Perhaps most intriguingly, HCD has been observed to substantially modulate gut microbiota composition, indicating a multifaceted mechanism of action that extends beyond their direct anti-inflammatory and antioxidant effects. This ability to simultaneously target multiple pathways involved in obesity, including inflammation, oxidative stress, and gut microbiome dysregulation—positions HCD as a promising candidate for prevention and treatment strategies. By offering a comprehensive approach to tackling the complex interplay of factors underlying this metabolic disorder, HCD represents a significant step forward in developing more effective obesity interventions.

Drawing from the variety of strategies described in recent literature, we propose a framework to better understand the current landscape and future directions of NP-based immunoengineering for obesity. These emerging approaches can be broadly grouped into three interconnected areas. First, immune cell reprogramming targets key immune cells such as MΦs, T cells, or dendritic cells within AT to resolve inflammation—for example, by using IL-4-loaded exosomes to shift MΦs toward an anti-inflammatory state. Second, metabolic receptor agonism leverages the precise targeting capabilities of NPs to activate pathways in fat cells that promote energy burning or regulate glucose, such as PPARγ, β3-adrenergic, GLP-1, and FGF21 receptors. Third, systemic crosstalk modulation aims to influence communication between organs, such as the gut, liver, and AT, often by delivering metabolites from the microbiota or hormones produced by the liver. Notably, systemic approaches typically act through circulating signals and do not require NPs to deeply penetrate tissues, which can simplify delivery but may also increase the risk of unintended effects in other organs. By categorizing the field in this way, we highlight important trade-offs: immune-focused strategies may offer long-lasting metabolic benefits, receptor-targeted systems can provide rapid control of blood sugar, and systemic modulators are easier to deliver but may be less specific. This perspective underscores the potential for next-generation NP therapies that combine these strategies to achieve precise, coordinated control over immune and metabolic processes in obesity.

## 4 Conclusions and future perspectives

The implications of obesity on the well-being of individuals are extensive and continue to increase without any indication of abatement. The existence of obesity and its associated metabolic irregularities can have a significant impact on the state of the immune system. Obesity induces a persistent state of inflammation, resulting in the polarization of MΦs towards a pro-inflammatory state and, subsequently, increased production of various inflammatory cytokines. The presence of immune cells and signals in WATs and BATs is crucial for maintaining tissue homeostasis, and their contribution cannot be overstated. They facilitate the efflux of lipids from white adipocytes while promoting high oxidation rates in brown adipocytes. Immune cells, such as eosinophils and alternatively activated MΦs, have been found to play important regulatory roles in maintaining metabolic homeostasis in both WATs and BATs. Ongoing research is focused on identifying additional immunological players involved in this process. If the underlying mechanism behind this regulatory function can be fully understood, immune regulation may be a promising therapeutic target for increasing energy expenditure and reducing weight gain, particularly using NPs. Notably, the number of immune cells present in both lean and obese BATs is significantly lower than in WATs, suggesting that BATs are relatively more resistant to inflammation induced by dietary factors. However, excess energy intake can still lead to increased tissue inflammation in BATs. In this discourse, we have highlighted some recent findings in this field, specifically focusing on MΦs, AT browning, and their implications in immunomodulation. This review aimed to describe the metabolic intercommunication between immune cells and brown and white adipocytes in ATs and investigate NPs to intervene in treating obesity. Moreover, it is imperative to determine the precise sequence of events that occur during obesity development. Additionally, BATs’ regulation by the immune system is not solely dependent on one specific immune cell type but requires extensive communication between multiple cell types.

Future research in the field of BATs and obesity management should focus on several key areas. Identifying specific effector molecules secreted by brown adipocytes for immune regulation is crucial for understanding BAT’s role in immune cell attraction and control. Elucidating the mechanisms regulating BAT activity in obesity could provide insights into potential therapeutic targets. The function of the sympathetic nervous system in BAT activation and its implications for obesity treatment warrant further investigation. Additionally, exploring how BAT activity changes during aging is essential for developing age-appropriate interventions. Translational research should focus on developing effective methods for activating and expanding BATs in clinical settings, including pharmacological, nutritional, and environmental approaches. By addressing these research priorities, the field can advance our understanding of BAT biology and its therapeutic potential in obesity-related disorders, potentially leading to more effective and targeted interventions for this global health challenge. Furthermore, many current experimental models are still unable to accurately simulate the complex interactions between NP and the biological processes associated with obesity. These models usually cannot fully represent human characteristics and are incapable of investigating the long-term effects of NP treatment in real-world conditions. Therefore, further research into specific molecular pathways and the development of more advanced clinical models are needed to fully evaluate the efficacy of these treatments and achieve reliable results.

## Figures and Tables

**Table 1:** A summary of commercially available obesity medications, the mechanisms of action, and side effects

	Drug	Brand names	Mechanism of action	Side effects	References
1	Orlistat	Xenical^®^, Alli^®^	Lipase inhibitor: Preventing the absorption of dietary fats in the intestines. The unabsorbed fat is excreted in the stool.	Bowel habits such as oily spotting, gas with discharge, urgent bowel movements, and fatty stools. Serious side effects can include liver damage, allergic reactions, and kidney stones.	[114, 115]
2	Phentermine-topiramate	Qsymia^®^	Sympathomimetic amine suppresses appetite, while topiramate is an anticonvulsant that enhances satiety and decreases appetite.	Dry mouth, constipation, insomnia, dizziness, and paresthesia. Serious side effects may include mood changes, suicidal thoughts, and increased heart rate.	[116, 117]
3	Naltrexone-bupropion	Contrave^®^	Opioid antagonists and bupropion are an aminoketone antidepressant. Together, they affect the central nervous system, reducing hunger and controlling cravings.	Nausea, constipation, headache, and insomnia. Serious side effects can include suicidal thoughts and increased blood pressure.	[116, 118]
4	Liraglutide	Saxenda^®^ Victoza^®^	GLP-1 receptor agonist which increases insulin secretion, decreases glucagon secretion, and slows gastric emptying to control blood sugar and reduce appetite.	Nausea, vomiting, diarrhea, and constipation. Serious side effects may include pancreatitis and thyroid tumors.	[119–123]
5	Semaglutide	Ozempic^®^ Wegovy^®^ Rybelsus^®^	GLP-1 receptor agonist enhances insulin secretion, reduces glucagon release, and slows gastric emptying, contributing to weight loss.	Nausea, diarrhea, vomiting, and constipation. Serious side effects can include pancreatitis and gallbladder disease.	[124–125]
6	Setmelanotide	Imcivree^®^	Melanocortin-4 receptor (MC4R) agonist that helps restore appetite control in patients with genetic obesity disorders.	Injection site reactions and skin hyperpigmentation. Serious side effects can include depression and suicidal ideation.	[126–128]
7	Tirzepatide	Zepbound^®^	Dual agonist for GLP-1 and GIP receptors, enhancing insulin secretion and reducing appetite.	Nausea, diarrhea, and decreased appetite. Serious side effects can include pancreatitis and gallbladder disease.	[129]

**Table 2:** Key biochemical and cellular markers reveal the inflammatory landscape of ATs

Biochemical factors	Description	References
Fibrinogen	Produced in the liver, regulated by IL-6 and TNF-α	[130]
C-reactive protein (CRP)	Systemic markers of inflammation increased rapidly due to immune activities	[131]
IL-6, TNF-α	Produced in WAT, an inflammatory mediator	[131]
UCP-1	Produced in BAT, mediates lipid oxidation from WAT	[132]
IL-10	Produced by M2 MΦs, M2 marker	[133]
miR-98	Negatively regulates IL-10 production	[134]
TLR4	Mediate LPS-induced pro-inflammatory signaling	[135]
IL-4	Promote ATM accumulation, induce macrophage polarization, and inhibit IL-6	[136, 137]
Osteopontin	Promote ATM proliferation	[138]
TRAF6, IRAK4, IKKβ	Downstream of TLR4 signaling	[92]
NF-κB	Inflammatory mediators promote M1 polarization	[134]
IL1β, COX2, IL12p40	Downstream of NF-κB signaling	[92]
HIF1α	Fatty acid oxidation and immune cell infiltration	[139]
TET1	Hypomethylation of hypoxia-induced gene promoters	[140]
Leptin, VEGF-A	Downstream of HIF1α/TET1 pathway under hypoxia	[140]
